# Treatment of Brachial Plexus Injury Following Transaxillary Thyroidectomy

**DOI:** 10.29252/wjps.10.3.114

**Published:** 2021-09

**Authors:** Hosseinali Abdolrazaghi, Javad Rahmati, Changiz Delavari, Hojjat Molaei

**Affiliations:** 1 Department of Hand & Reconstructive Surgery, Sina Hospital, School of Medicine, Tehran University of Medical Sciences, Tehran, Iran; 2 Department of Plastic & Reconstructive Surgery, Razi Hospital, School of Medicine, Tehran University of Medical Sciences, Tehran, Iran; 3 Department of Plastic & Reconstructive Surgery, IKHC, School of Medicine, Tehran University of Medical Sciences, Tehran, Iran

**Keywords:** Brachial Plexus Injury, Transaxillary Thyroidectomy

## Abstract

Minimally invasive surgeries are widespread and technically enhancing. Thyroidectomy is a common surgery and non-invasive adjustments make it more interesting. Neighbor neurovascular bundles need to be protected during minimally invasive thyroidectomy. A 15 yr old female who underwent minimally invasive thyroidectomy due to nodule, had presented with upper brachial plexus injury, without proper recovery despite physiotherapy cessions. She was operated in 2 stage reconstructive surgeries. First, musculocutaneous nerve innervated by 2 branches of median and ulnar nerves. Then, in a compound operation, axillary nerve innervated by long head branch of triceps nerve and suprascapular nerve by accessory nerve. She gained good function of upper limb. Minimally invasive operations in head and neck area can be disastrous, if surgeons do not consider anatomical points. Brachial plexus reconstructive surgeries are complicated operations to preserve hand functions following iatrogenic injuries.

## INTRODUCTION

The brachial plexus has the most complex structure among peripheral nerves, and brachial plexus injury (BPI) in adults remains one of the most challenging issues in microsurgery. The odds of nerve repair decrease by 1% every six days after nerve injury and delayed surgeries such as tendon transfer, muscle transfer, and the free flap should be undertaken, unless immediate interventions would had been considered^1^. Sometimes, unwanted iatrogenic complications can change to catastrophes with deep effects. 

## CASE PRESENTATION

A 15-year-old female underwent minimally invasive transaxillary thyroidectomy due to a diagnosed right thyroid lobe nodule. Immediately after the surgery, she experienced shoulder and elbow paralysis and hoarseness. Examination indicated severe upper trunk plexopathy, brachial plexus injury with upper trunk rupture, and right recurrent laryngeal nerve injury. She was referred to Sina Hospital, Tehran, Iran in winter 2020 after 30 sessions of unsuccessful physiotherapy to reconstruct. 

**Fig. 1:**
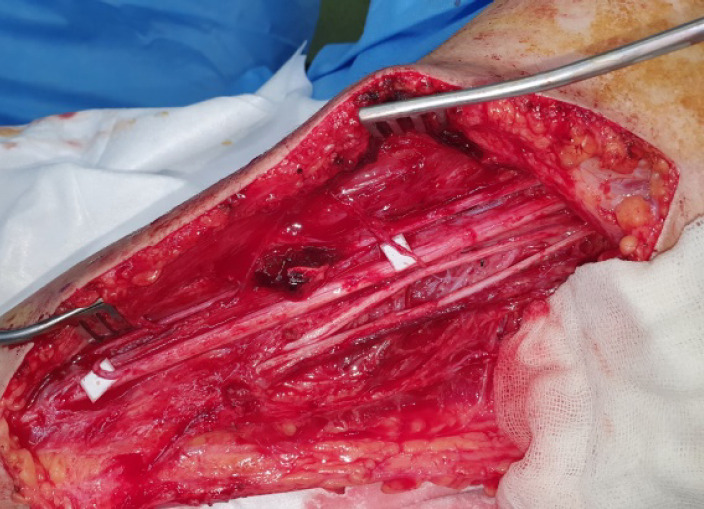
First surgery in supine position. Surgery scene as demonstrated by white backgrounds nerve fascicles transferred from the ulnar nerve to the biceps brachii of the musculocutaneous nerve and from the median nerve to the brachialis branch of the musculocutaneous nerve

All the procedures and possible outcomes discussed and she and her parents signed informed consents to start reconstructive treatments. During first surgery, through medial proximal right arm incision, nerve fascicles transferred from the ulnar nerve to the biceps brachii of the musculocutaneous nerve and from the median nerve to the brachialis branch of the musculocutaneous nerve (Figure 1). 

**Fig. 2:**
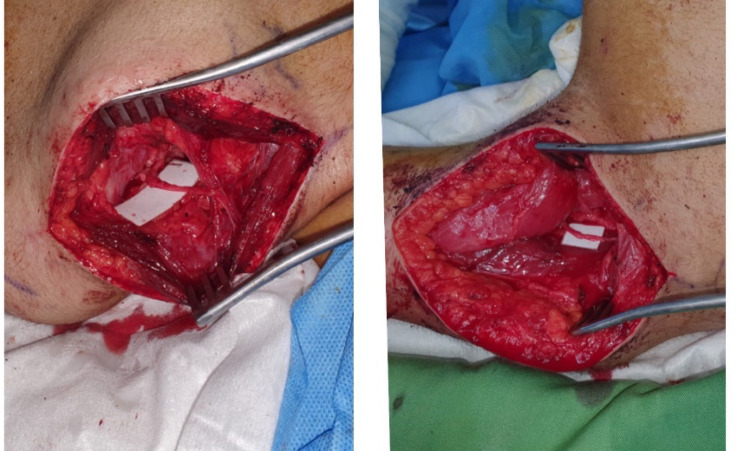
Second surgery scene in prone position

 About three weeks later, in the second surgery, via posterior approach, with a transverse incision in upper scapula border the spinal accessory nerve was transferred to the suprascapularis nerve under the microscope coaptation (Figure 2 right). Next, through an incision of posterior arm, the radial branch of the long head of the triceps brachii was identified using the nerve stimulator and eliciting triceps muscle contraction, and this nerve was transferred to the anterior branch of the axillary nerve under the microscope (Figure 2 left). She had been under strict nerve stimulation and physiotherapy for about 4 months and fortunately gained most of shoulder and elbow activities accordingly. 

Right: spinal accessory nerve was transferred to the suprascapularis nerve. Left: branch of the long head of the triceps brachii was transferred to the anterior branch of the axillary nerve.

## DISCUSSION

The incidence of transient or permanent recurrent laryngeal nerve injury and brachial plexus injury in transaxillary thyroidectomy has been reported to be 1.23%, 0.27%, and 0.2%, respectively, in some reports^1^. 

In plexus injuries, transfer of the spinal accessory nerve to the suprascapularis nerve restores shoulder function up to 80% and muscle strength reaches the M3 level^2^. Shoulder abduction can improve up to 124 degrees, especially if the above transfer is accompanied by the transfer of the radial branch of the long head of the triceps brachii to the anterior branch of the axillary nerve^3^. With nerve fascicle transfer from the ulnar nerve to the biceps branch of the musculocutaneous nerve and from the median nerve to the brachialis branch of the musculocutaneous nerve, the patient attains grade 3 (M3) muscle strength in 75%-100% of cases, although sometimes these nerve transfers produce co-contraction (2). 

In the patient discussed, we avoided the above and below clavicle approaches and plexus exploration in nerve scar and graft tissue for the following reasons: (i) Clavicle osteotomy and other injuries could occur if we tried to explore the scar tissue in this patient,; (ii) The patient would need a sural nerve resection and an additional incision; (iii) Sural nerve graft in the affected plexus would have lengthened the surgery and necessitated two transplants for each injured nerve; and eventually (iv) in nerve transfer, the distance between the donor and the receptor nerves is short, and therefore, nerve recovery occurs more quickly than in the nerve graft^4^. Therefore, the final result of such complicated catastrophe was acceptable and promising patient to continue her physiotherapy sessions. 

## CONCLUSION

Return of elbow and shoulder movements is a priority in adult brachial plexus injuries, and timely diagnosis and referral of these patients reduce future morbidities. The best type of microscopic surgery can be chosen for the patient with careful examination and the presence of a treatment team.

## CONFLICT OF INTEREST

The authors declare that there is no conflict of interests.
